# Expression of the Cystatin SN (CST1) in Breast Cancer and Its Association With Clinical Characteristics: A Cross-Sectional Study

**DOI:** 10.7759/cureus.109177

**Published:** 2026-05-19

**Authors:** Sumaya Najnin, Neelufar Sultana Chowdhury, Raisa Ferdous, Pronati Paul, Jahirul Islam, Mohammad Hafizur Rahman

**Affiliations:** 1 Department of Biochemistry, Gonoshasthaya Samaj Vittik Medical College, Dhaka, BGD; 2 Department of Biochemistry, Dhaka Medical College, Dhaka, BGD; 3 Department of Biochemistry, Barrister Rafiq-Ul-Huq Medical College, Dhaka, BGD; 4 Department of Physiology, Delta Medical College, Dhaka, BGD; 5 Department of Hepatology, Bangladesh Medical University, Dhaka, BGD

**Keywords:** bangladesh, biomarker, breast cancer, cst1, gene expression, next-generation sequencing

## Abstract

Background

Breast cancer is a major global health concern with increasing incidence and mortality, particularly in low- and middle-income countries such as Bangladesh. Cystatin SN (CST1) has emerged as a potential oncogenic biomarker; however, its expression and clinical relevance remain underexplored in Bangladeshi populations. This study aimed to evaluate CST1 expression in tumor and adjacent normal breast tissues and to assess its association with the clinical characteristics of breast cancer patients.

Methods

A hospital-based cross-sectional study was conducted at Dhaka Medical College, Bangladesh, from January 2023 to June 2023. A total of 34 adult female patients (aged 25-70 years) with histologically confirmed, treatment-naïve breast cancer were recruited via purposive sampling. Paired tumor and adjacent normal tissue samples were collected following a modified radical mastectomy. CST1 gene expression was measured via next-generation sequencing. The data were analyzed via SPSS version 26 (IBM Corp., Armonk, NY, USA). The Wilcoxon signed-rank test was used to compare gene expression between paired samples, and Spearman's correlation coefficient was applied to assess associations with clinical variables.

Results

The mean age of the participants was 47.41 ± 12.19 years. CST1 gene expression was significantly greater in tumor tissue than in adjacent normal tissue (median -0.068 vs -1.988, p = 0.035). A statistically significant positive association was observed between CST1 expression and a family history of breast cancer (rho = 0.267, p = 0.027). In contrast, CST1 expression was significantly negatively associated with contraceptive use (rho = -0.437, p < 0.001) and weakly negatively associated with breastfeeding history (rho = -0.240, p = 0.049). No significant associations were observed between CST1 expression and age, body mass index, parity, or age at menarche.

Conclusion

CST1 gene expression is significantly elevated in tumor tissue and is associated with selected clinical factors, suggesting its potential relevance as a molecular marker in breast cancer. Further large-scale and longitudinal studies are needed to establish its clinical and prognostic significance.

## Introduction

Breast cancer continues to be the most commonly diagnosed malignancy among women globally and remains a major contributor to cancer-related deaths. Data from the GLOBOCAN 2022 report by the International Agency for Research on Cancer (IARC) indicate that nearly 2.30 million new cases and 666,103 deaths occurred worldwide, highlighting its position as the most prevalent cancer among females [1]. Asia contributed the largest share of cases (985,817), with a global age-standardized incidence rate of approximately 48 per 100,000 women [2]. Projections suggest that by 2050, the number of annual cases may increase to 3.55 million, with the number of deaths reaching 1.14 million, underscoring the urgent need for improved strategies for early detection, molecular characterization, and targeted therapy [1].

Breast cancer is a biologically heterogeneous disease characterized by genetic and epigenetic alterations that result in distinct molecular subtypes, including luminal A, luminal B, human epidermal growth factor receptor 2 (HER2)-enriched, and basal-like subtypes [3]. These subtypes differ in prognosis and therapeutic response, making molecular profiling essential for clinical decision-making. Established biomarkers, such as estrogen receptor (ER), progesterone receptor (PR), human epidermal growth factor receptor 2 (HER2), BRCA1/2, and TP53, are routinely used for risk stratification and treatment planning [4]. Advances in next-generation sequencing (NGS) have further enhanced the understanding of tumor biology, demonstrating that mutations in TP53 and BRCA1 are associated with poorer outcomes, particularly in aggressive subtypes such as triple-negative breast cancer [5].

Among emerging molecular markers, cystatin SN (CST1) has gained increasing attention for its oncogenic role. CST1 is a secretory protein belonging to the type 2 cystatin superfamily and acts as an inhibitor of cysteine proteases such as cathepsin B, which are involved in tumor invasion and extracellular matrix degradation [6]. The overexpression of CST1 has been reported in multiple cancers, including gastric, colorectal, pancreatic, and breast cancers, where it promotes cell proliferation, inhibits apoptosis, and facilitates epithelial-mesenchymal transition [7]. Mechanistically, CST1 is involved in key signaling pathways, such as the phosphoinositide 3-kinase (PI3K)/protein kinase B (AKT) and IL-6/Janus Kinase (JAK)-signal transducer and activator of transcription 3 (STAT3) pathways, which contribute to tumor growth, invasion, and treatment resistance [8,9].

In breast cancer specifically, CST1 expression has been shown to be significantly elevated in tumor tissues compared with adjacent normal tissues. Dai et al. reported approximately fourfold upregulation of CST1 in breast cancer using the Cancer Genome Atlas (TCGA) data, with validation in clinical samples at both the mRNA and protein levels [6]. Importantly, lower CST1 expression was associated with significantly improved overall and disease-free survival, and multivariate analysis identified CST1 as an independent prognostic factor [6]. Functional studies further demonstrated that CST1 enhances tumor cell proliferation, migration, and invasion, whereas its suppression reduces tumor growth [6]. Recent evidence also suggests a role for CST1 in tamoxifen resistance in ER-positive breast cancer, extending its clinical relevance [10].

The comparison of gene expression between tumor and adjacent normal tissues is fundamental for biomarker discovery, as it provides insights into tumorigenesis and potential therapeutic targets [3]. However, although CST1 has been studied in various cancers, its association with the clinical and pathological features of patients with breast cancer has not been fully explored, particularly in diverse populations.

The burden of breast cancer is especially concerning in low- and middle-income countries (LMICs), where mortality rates are disproportionately high because of late diagnosis and limited access to advanced diagnostics [11]. In Bangladesh, breast cancer accounts for approximately 32.8% of female cancers and nearly 69% of cancer-related deaths, with most cases being diagnosed at advanced stages [12]. Delayed diagnosis is common, with nearly half of patients experiencing delays exceeding three months [13]. Despite this burden, molecular research using advanced genomic tools remains limited, and to the best of our knowledge, no study has yet evaluated CST1 expression in Bangladeshi breast cancer patients [14]. Therefore, the present study aimed to evaluate CST1 gene expression in tumor and normal breast tissues and to assess its association with the clinical characteristics of breast cancer patients in Bangladesh.

## Materials and methods

Study design and setting

A hospital-based cross-sectional study was carried out at the Department of Biochemistry, Dhaka Medical College, Dhaka, Bangladesh, in collaboration with the Institute for Population and Precision Health, Department of Public Health Sciences, Biological Sciences Division, University of Chicago, Chicago, USA. The study was conducted over a six-month period from January 2023 to June 2023.

Study population

The study population comprised adult female patients with histologically confirmed breast cancer who attended the Department of General Surgery (Breast Clinic), Dhaka Medical College Hospital, during the study period. The diagnosis of breast cancer was established on the basis of clinical evaluation and confirmed by histopathological examination, which was supported by fine needle aspiration cytology (FNAC) and relevant imaging findings where indicated. Patients aged between 25 and 70 years were included in the study. Only newly diagnosed, treatment-naïve patients were enrolled. Patients with a history of any other malignancy, those who had previously received chemotherapy, radiotherapy, or surgical treatment for breast cancer, and those unwilling to participate or provide informed consent were excluded from the study. Relevant clinical information, including reproductive history and family history of breast cancer, was collected through structured interviews and a review of clinical records.

Sampling procedure

A nonprobability purposive sampling technique was employed to recruit participants. Eligible patients who presented to the breast clinic during the study period were approached consecutively and screened according to predefined inclusion and exclusion criteria. Patients who fulfilled the eligibility criteria and provided written informed consent were enrolled in the study.

Sample size determination

As there were limited prior Bangladeshi data regarding CST1 gene expression in breast cancer tissues, a formal sample size calculation based on paired gene expression differences or correlation coefficients could not be reliably performed. Therefore, all eligible treatment-naïve breast cancer patients presenting during the study period and fulfilling the inclusion criteria were consecutively enrolled. A total of 34 participants were included in the final analysis. The present study was considered exploratory in nature.

Data collection procedures

Data were collected through face-to-face interviews and a review of clinical records via a pretested semistructured data collection sheet. Patients attending the surgical department were initially evaluated through detailed history, clinical examination, and relevant diagnostic investigations. After confirmation of eligibility, the participants were informed about the objectives and procedures of the study, and written informed consent was obtained. Sociodemographic and clinical data were collected through structured interviews. Following modified radical mastectomy (MRM), approximately 0.5 cm³ of tumor tissue and 0.5 cm³ of adjacent normal breast tissue were collected from each participant in separate preservative-containing Eppendorf tubes. The collected tissue samples were preserved in a DNA/RNA stabilizing solution and maintained under controlled temperature conditions prior to transportation for molecular analysis. All data collection procedures were conducted by the investigators to ensure consistency and accuracy.

Measurement of the CST1 gene expression

The expression of CST1 was analyzed via next-generation sequencing (NGS) technology [15]. RNA was extracted from both tumor and adjacent normal tissue samples using the Quick-DNA/RNA™ Microprep Plus Kit (Zymo Research, CA, USA) according to the manufacturer’s protocol. RNA quality and concentration were assessed using a NanoDrop 1000 spectrophotometer (Thermo Fisher Scientific, USA) and further validated through electrophoretic analysis using an Agilent Bioanalyzer (Agilent Technologies, USA). Library preparation and targeted RNA sequencing were performed using the Twist RNA Library Preparation Kit (Twist Bioscience, CA, USA) following the manufacturer’s instructions. Sequencing data were processed and analyzed using standard bioinformatics pipelines. Raw sequencing reads underwent quality control procedures and were aligned to the human reference genome (GRCh38) using widely accepted alignment tools such as HISAT2 or STAR. Gene expression quantification was performed, and normalized transcript levels were calculated as transcripts per million (TPM) to account for sequencing depth and gene length variations. For downstream statistical analysis, normalized TPM values were log2 transformed after the addition of a small constant to reduce skewness and improve comparability across samples. Consequently, some transformed expression values appeared as negative values in the statistical summaries. The average sequencing depth was maintained at approximately 20-30 million reads per sample to ensure reliable transcript quantification. All laboratory analyses were performed by a researcher who was blinded to the clinical characteristics of the participants to minimize measurement bias. Quality control procedures were applied at multiple stages, including assessment of read quality, removal of low-quality reads and adapters, and validation of sequencing performance metrics prior to downstream analysis.

Operational definitions

Normal breast tissue was defined as tissue obtained at least 2 cm away from the tumor margin, appearing macroscopically normal (soft, yellowish, and without any suspicious lesions), and confirmed to be free of malignancy based on intraoperative assessment and postoperative histopathological evaluation, where applicable. Tumor tissue was defined as histologically confirmed malignant breast tissue obtained from the primary tumor mass [16]. Body mass index (BMI) was calculated as weight in kilograms divided by height in meters squared and categorized according to World Health Organization criteria [17].

Statistical analysis

The data were entered, cleaned, and analyzed using the Statistical Package for the Social Sciences (SPSS) version 26 (IBM Corp., Armonk, NY, USA). Continuous variables were assessed for normality using the Shapiro-Wilk test. As the CST1 gene expression data were not normally distributed, continuous variables were summarized as medians with interquartile ranges (IQRs), whereas normally distributed variables were expressed as means ± standard deviations (SDs). Categorical variables are presented as frequencies and percentages. Comparison of CST1 gene expression between paired tumor and adjacent normal breast tissue samples was performed using the Wilcoxon signed-rank test. The relationships between CST1 gene expression and clinical variables were evaluated using Spearman’s rank correlation coefficient (rho). For dichotomous variables (e.g., family history of breast cancer, contraceptive use, and breastfeeding history), responses were coded as binary variables (0 = no, 1 = yes) to facilitate correlation analysis. This approach was used to explore monotonic associations in this exploratory study. The strength of correlation was interpreted as poor (0.00-0.29), fair (0.30-0.59), moderate (0.60-0.79), or very strong (≥0.80) [18]. A p-value less than 0.05 was considered statistically significant. As this was an exploratory study with a limited sample size, formal correction for multiple comparisons was not applied, and the findings should therefore be interpreted cautiously.

Ethical considerations

Ethical approval for the study was granted by the Ethical Review Committee of Dhaka Medical College (Memo No. ERC-DMC/ECC/2022/442). The study adhered to the ethical standards outlined in the Declaration of Helsinki. All the participants were clearly informed about the objectives, procedures, and voluntary nature of their participation. Written informed consent was obtained prior to inclusion in the study. Participant confidentiality and anonymity were rigorously protected, and individuals were informed of their right to withdraw from the study at any time without any penalty or adverse consequences.

## Results

Table 1 summarizes the baseline sociodemographic and clinical characteristics of the participants. The study population was predominantly middle-aged, with a mean age of 47.41 ± 12.19 years, and the majority of participants were aged ≤50 years (26, 76.47%). In terms of nutritional status, more than half of the participants had a normal body mass index (BMI) (18, 52.94%), whereas the remaining 47.06% (16) were overweight. The mean BMI of the participants was 21.07 ± 1.17 kg/m². In terms of reproductive and hormonal factors, early menarche was reported in 58.82% (20) of the participants. A high proportion of participants had a history of contraceptive use (29, 85.29%). A family history of breast cancer was present in 12 (35.29%) of the patients. Most of the participants were multiparous (27, 79.41%), and a large majority had a history of breastfeeding (28, 82.35%).

**Table 1:** Sociodemographic and clinical characteristics of the participants (n = 34).

Variable	Category	n (%)
Age	≤50 years	26 (76.47)
	>50 years	8 (23.53)
	Mean ± SD	47.41 ± 12.19
BMI	Normal	18 (52.94)
	Overweight	16 (47.06)
	Mean ± SD	21.07 ± 1.17
Early menarche	Yes	20 (58.82)
	No	14 (41.18)
Contraceptive use	Yes	29 (85.29)
	No	5 (14.71)
Family history of breast cancer	Yes	12 (35.29)
	No	22 (64.71)
Parity	Multipara	27 (79.41)
	Others	7 (20.59)
Breastfeeding history	Yes	28 (82.35)
	No	6 (17.65)

The expression of the CST1 gene in tumor and adjacent normal breast tissues is presented in Table 2. The median expression level of the CST1 gene was markedly greater in tumor tissue than in normal tissue. Specifically, the median (IQR) level of CST1 expression in normal tissue was -1.988 (-2.58 to 0.36), whereas in tumor tissue, it was -0.068 (-1.57 to 1.69). This difference was statistically significant (p = 0.035).

**Table 2:** Expression of the CST1 gene in tumor and normal breast tissues (n=34). Wilcoxon signed-rank test was used for comparison. Z = test statistic value, IQR: interquartile range, CST1: cystatin SN.

Variable	Median (IQR)	Test statistic	p-value
CST1 expression (normal tissue)	-1.988 (-2.58 to 0.36)	Z = -2.10	0.035
CST1 expression (tumor tissue)	-0.068 (-1.57 to 1.69)		

The associations between CST1 gene expression and selected clinical variables are presented in Table 3. A statistically significant positive association was observed between CST1 gene expression and a family history of breast cancer (rho = 0.267, p = 0.027). Conversely, CST1 gene expression demonstrated a statistically significant negative association with contraceptive use (rho = −0.437, p < 0.001) and a weak negative association with breastfeeding history (rho = −0.240, p = 0.049). No statistically significant associations were found between CST1 gene expression and age at menarche (rho = 0.023, p = 0.855), age (rho = −0.231, p = 0.058), BMI (rho = −0.009, p = 0.942), or parity (rho = 0.007, p = 0.932).

**Table 3:** Associations of CST1 gene expression with clinical variables (n = 34). Spearman's rank correlation test was used. rho = correlation coefficient, CST1: cystatin SN.

Variable	rho-value	p-value
Family history of breast cancer	0.267	0.027
Contraceptive use	-0.437	<0.001
Breastfeeding history	-0.240	0.049
Age at menarche (years)	0.023	0.855
Age	-0.231	0.058
BMI	-0.009	0.942
Parity	0.007	0.932

## Discussion

In the present study, we aimed to evaluate CST1 gene expression in tumor and adjacent normal breast tissues and to assess its association with clinical characteristics of breast cancer patients. Our findings revealed significantly higher CST1 expression in tumor tissue compared with normal tissue, suggesting a potential role in tumor biology. CST1 expression was significantly associated with selected clinical variables, including family history, contraceptive use, and breastfeeding, whereas no significant relationships were observed with age, BMI, parity, or age at menarche.

One of the key findings of this study was significantly higher CST1 expression in tumor tissue than in adjacent normal breast tissue, which is consistent with previous molecular evidence. Dai et al. reported marked upregulation of CST1 at both the mRNA and protein levels in breast cancer tissues, with transcriptomic analyses showing substantially greater expression in tumor samples than in normal breast tissue [6]. Similar findings were described by Liu et al., who demonstrated preferential overexpression of CST1 in estrogen receptor-positive breast cancer cells and showed that CST1 promotes tumor proliferation through the PI3K/protein kinase B (AKT)/ERα signaling loop, whereas CST1 knockdown suppresses cell cycle progression and tumor growth [8]. Biologically, CST1 is a member of the type 2 cystatin family and functions as an inhibitor of cysteine proteases, particularly cathepsin B [19]. Because cathepsin B participates in extracellular matrix degradation and tumor microenvironment remodeling, CST1 dysregulation may be associated with tumor invasion, progression, and metastatic potential [9]. Experimental evidence also suggests that CST1 is involved in epithelial-mesenchymal transition and the regulation of senescence-related pathways, further suggesting a possible oncogenic role [7]. In addition, CST1 overexpression has been implicated in tamoxifen resistance and metastatic behavior in ER-positive breast cancer, which may further support its potential clinical relevance [10].

Another important observation was the positive association between CST1 expression and a family history of breast cancer. Family history is a well-established marker of hereditary susceptibility, and germline mutations in genes, such as BRCA1, BRCA2, ATM, CHEK2, and PALB2, contribute substantially to familial breast cancer risk [20]. A previous molecular study revealed that familial breast cancers may demonstrate distinct expression profiles and clustering of molecular subtypes within affected families, suggesting that an inherited genetic background can influence tumor transcriptional behavior [21].

The inverse associations of CST1 expression with breastfeeding and contraceptive use should be interpreted cautiously but remain noteworthy. Breastfeeding has long been considered protective against breast cancer through mechanisms including reduced cumulative estrogen exposure, enhanced terminal differentiation of the mammary epithelium, and epigenetic regulation that may limit malignant transformation [22]. Meta-analytic evidence indicates that a longer duration of breastfeeding is associated with a measurable reduction in breast cancer risk [23]. The negative association observed here may therefore reflect a potentially protective hormonal environment that may be associated with lower CST1 expression. In contrast, the inverse relationship with contraceptive use is more difficult to interpret. Although pooled evidence has suggested a modest increase in breast cancer risk among hormonal contraceptive users [24], the present findings may reflect differences in contraceptive formulation, duration of exposure, menopausal status, or unmeasured confounding factors.

In the present study, no significant associations were found between CST1 expression and age at menarche, age, BMI, or parity. This is not entirely unexpected, as these factors are more consistently associated with breast cancer risk or subtype distribution than with the expression of a single gene in established tumors [25-27]. The absence of significant associations may indicate that CST1 expression is more strongly influenced by intrinsic tumor biology and inherited molecular susceptibility than by broader demographic or reproductive characteristics. Overall, these findings suggest that CST1 may represent a biologically relevant marker in breast cancer.

## Conclusions

CST1 gene expression was significantly higher in tumor tissue than in adjacent normal breast tissue, suggesting its potential involvement in breast cancer biology. Significant associations were observed with selected clinical variables, including family history, contraceptive use, and breastfeeding, whereas no significant relationships were found with age, BMI, parity, or age at menarche. These findings indicate that CST1 may be relevant as a molecular marker in breast cancer. However, larger studies with longitudinal follow-up are needed to further clarify its clinical and prognostic significance.
